# Living Well with Pollution? The Impact of the Concentration of PM_2.5_ on the Quality of Life of Patients with Asthma

**DOI:** 10.3390/ijerph16142502

**Published:** 2019-07-13

**Authors:** Monika Ścibor, Andrzej Galbarczyk, Grazyna Jasienska

**Affiliations:** Department of Environmental Health, Faculty of Health Sciences, Jagiellonian University Medical College 20 Grzegorzecka St., PL 31531 Krakow, Poland

**Keywords:** environmental health, PM_2.5_, quality of life, AQLQ, asthma

## Abstract

While the negative influence of environmental pollution on the respiratory system is well established, especially for people with bronchial hyper-reactivity, the impact of particulate matter on quality of life in asthma patients is not well understood. Three hundred adult asthma patients were recruited for a study; for each patient, the daily concentrations of particulate matter of 2.5 µm or less in diameter (PM_2.5_) were recorded from air quality monitoring stations. The study was conducted over two weeks. After two weeks, the patients filled out the Asthma Quality of Life Questionnaire (AQLQ), evaluating the quality of their lives throughout the monitored period. Patients exposed to a higher concentration of PM_2.5_ had significantly lower AQLQ scores. Every 10 µg/m^3^ of an increase in the concentration of PM_2.5_ resulted in a decrease of the AQLQ score by 0.16. All domains of quality of life (symptoms, activity limitations, emotional functioning, and environmental stimuli) assessed in the questionnaire were negatively affected by PM_2.5_. These findings provide an important argument in favor of educating physicians and patients and raising awareness about the detrimental health effects of air pollution. Improving the quality of life of people with asthma requires an immediate and substantial reduction of air pollution.

## 1. Introduction

Bronchial asthma is one of the most frequently occurring chronic diseases [1]. Like every chronic disease, it poses a major problem not only in medical terms but also in social and economic terms [2,3,4,5]. As a long-term, incurable disease, it is a source of hardship and limitations for many patients pursuing their everyday activities, negatively impacting the quality of their lives.

The negative influence of particulate matter (PM) on the respiratory system has been known for a long time, especially in the case of patients with bronchial hyper-reactivity [6,7]. However, the evaluation of the impact of particulate matter on the quality of life of patients with bronchial asthma has not yet been widely analyzed. To the best of our knowledge, only limited evidence exists to suggest that fine particles measuring less than 10 µm (PM_10_) has an impact on the quality of life of patients with bronchial asthma [8], with no conclusive confirmation of the detrimental connection between the quality of life and the concentration of PM_10_. No studies were conducted concerning particulate matter of 2.5 µm or less in diameter (PM_2.5_).

PM_2.5_ (particles which pass through a size-selective inlet with a 50% efficiency cut-off at 2.5 μm aerodynamic diameter) have been classified by International Organization for Standardization as the “high-risk” respirable fraction [9]. PM_2.5_, also known as respirable dust, is an exceptionally harmful fraction because it penetrates the smallest bronchioles and the alveoli. Therefore, it may interfere with gas exchange inside the lungs and trigger or exacerbate respiratory diseases. Moreover, some fraction of inhaled PM_2.5_ (most likely the fraction of particles under 0.1 micrometer) may be capable to translocate into blood vessels and then spread with the blood to various tissues and organs [10].

According to WHO, the maximum 24 h average concentration of PM_2.5_ should not exceed 25 µg/m^3^. At the same time, while considering PM_2.5_ as “the most harmful among all atmospheric pollutants”, WHO states there is no safe level, below which we can be sure of the lack of negative effects for human health [11].

In studies conducted to date, researchers evaluated the impact of air pollutants mainly on the aggravation of symptoms of asthma [10,12,13]. However, an evaluation of the relationship between the environment and the quality of life of patients suffering from bronchial asthma has not yet been the subject of a sufficient number of studies. Results of such studies would be important in planning and implementing preventive measures taken in the area of public health, and would lead to the well-being of patients with bronchial asthma. The aim of our study was the evaluation of the impact of PM_2.5_ air pollution on the self-assessed quality of life of patients with bronchial asthma.

## 2. Materials and Methods

Patients with bronchial asthma (*n* = 349) who were treated in two allergy treatment clinics in Krakow, Poland were recruited for the study between June 2013 and May 2015. The diagnosis of asthma was confirmed by a physician (allergy specialist) according to the current guidelines [14]. Participants of the study were adult patients (>18 years old) with partially controlled asthma and were inhabitants of Krakow, in Poland. They declared that they would not leave the city throughout the 14 days of observation. Partly-controlled asthma means that patient had been experiencing one or two of the following features of asthma in the past four weeks: daytime symptoms more than twice a week, night waking due to asthma, rescue medication needed for symptom relief more than twice a week, activity limitation due to asthma [14].

During the first visit, the physician conducted the interview and recorded information about the date of birth, place of residence, education, employment, smoking, having pets, and types of asthma treatment. During the first visit, the patients also signed the informed consent for participation in the study and received a journal for recording the number of hours spent outside and the name of streets that they used for walking or travel each day. Each patient recorded this information every day for two weeks.

For each patient, we recorded the daily concentrations of PM_2.5_ for each day of observation from all available air quality monitoring stations of the Voivodeship Inspectorate of Environmental Protection in Krakow. Subsequently, each patient was assigned to the station closest to their declared outdoor place of stay, to estimate exposure to PM_2.5_ during the study. During the second visit (after 14 days) the patients’ quality of life was evaluated using the Standardised Asthma Quality of Life Questionnaire (AQLQ) for adults [15,16,17], which has been commonly used for measuring the asthma-specific quality of life [16,18]. The patients filled out the AQLQ, consisting of 32 questions (concerning the last 14 days). Questions were grouped into four domains: symptoms (12 questions), activity limitations (11 questions), emotional functioning (five questions), and environmental stimuli (four questions).

The patients marked their answers to each question on a seven-degree Likert scale, on which 7 meant “no limitations”, and 1 meant “total limitations” in performing particular activities. The overall score of the AQLQ total was the average of all 32 answers, and the score for a particular domain was the average value for the questions within this domain.

For analysis, we used the data from 300 patients aged 20–80 years old (mean 53, standard deviation/SD/15.3): 145 women (48.3%) and 155 men (51.7%). Forty-nine patients (14%—23 women and 26 men, aged 20–76 years old; mean 51, SD 13.3) were excluded due to leaving the city throughout observation or not filling out/losing the journal of observations. The study was conducted with the approval of the Committee of Bioethics of the Jagiellonian University (number KBET/167/B/2012).

Data were presented as means with standard deviations (SD) or the frequency with percentage distribution, respectively to its measurement scale. Two-week mean PM_2.5_ concentrations were additionally characterized by their quartile distribution. PM_2.5_ concentration was analyzed both as a continuous variable and as a categorical variable, according to its quartile distribution. Potential confounders of PM_2.5_ exposure and AQLQ, such as gender, education, active smoking, employment status, and having pets at home, were controlled in analyses. Associations between these nominal variables and PM_2.5_ categories were verified using a chi-square test or the exact Fisher test when appropriate. Differences in the age of patients exposed to different concentrations of PM_2.5_ categories (different quartiles) were tested using one-way ANOVA (equality of variances has been verified with Levene’s test and normal distribution in subgroups with the Kołmogorow–Smirnow test). The nonparametric trend in AQLQ values across PM_2.5_ categories was checked.

The potential impact of airborne PM_2.5_ on the quality of life of asthma patients (AQLQ total and its four domains) was estimated with linear regression models using the two-week mean of PM_2.5_ concentrations both as a categorical variable (according to quartile distribution and by the value of 25 µg/m^3^—the 24 h threshold suggested by WHO, which we used since there is no two-week guideline value) and as a continuous variable with a unit of 10 µg/m^3^, both adjusted to age (in years), gender, and university education (yes versus no) smoking (yes versus no) and season of measurement (cold: October to April, versus warm: May–September). Among the variables considered as potential confounders, only age and university education were significantly associated with AQLQ total and its domains in univariate analysis. Gender, smoking, and season of measurement were a priori added to models.

The AQLQ total was classified into three categories: good quality of life (score: 6–7), reduced quality of life (score: 4–5), and poor quality of life (score: 1–3), ordered from good as “1” to poor as ”3”. PM_2.5_ exposure level (both as a categorical and continuous variable) was used to assess its impact on poor asthma quality of life using ordered logistic regression models adjusted to age, age^2^, gender, and university education. The same analyses were conducted for all four AQLQ domains, as four separate tests. The proportional odds assumption was verified using the Brant test. The term age^2^ was added as age had not complied with the proportional odds assumption. The ordered logistic regression models were not adjusted for smoking as it did not affect the risk of poorer asthma life quality. Besides, only 31 (10%) patients were smokers. These patients were attempting to quit smoking and they reported smoking no more than between one to three cigarettes per day at the time of observation. All tests were two-tailed, and significance was set at *p* < 0.05. All analyses were performed using STATA/IC 13.1 (StataCorp LP, College Station, TX, USA).

## 3. Results

PM_2.5_ exposure (mean in a two-week period) averaged 45.1 µg/m^3^ (median 46; range: 9.6–90.1 µg/m^3^) and only 75 (25%) patients were exposed to values lower or equal to 25 µg/m^3^, the 24 h threshold recommended by WHO. PM_2.5_ measurements were equally distributed across seasons. There were no statistically significant differences between patients in the number of hours spent outdoors. In general, all declared that they spend 2–3 h a day outdoors, on average.

Groups of patients with different average PM_2.5_ exposure levels (classified by quartiles of PM_2.5_) did not differ significantly in gender, education level (university versus lower), employment status, or keeping pets at home. Nevertheless, patients exposed to higher levels of PM_2.5_ were older and the studied subgroups were significantly different according to active smoking prevalence (Table 1). The mean asthma quality of life (AQLQ total) in the studied group was 5.0 (range: 2–7). Declining trends in total and specific domains of asthma quality of life were observed across increasing levels of airborne PM_2.5_ exposure (Table 1).

**Figure 1:**
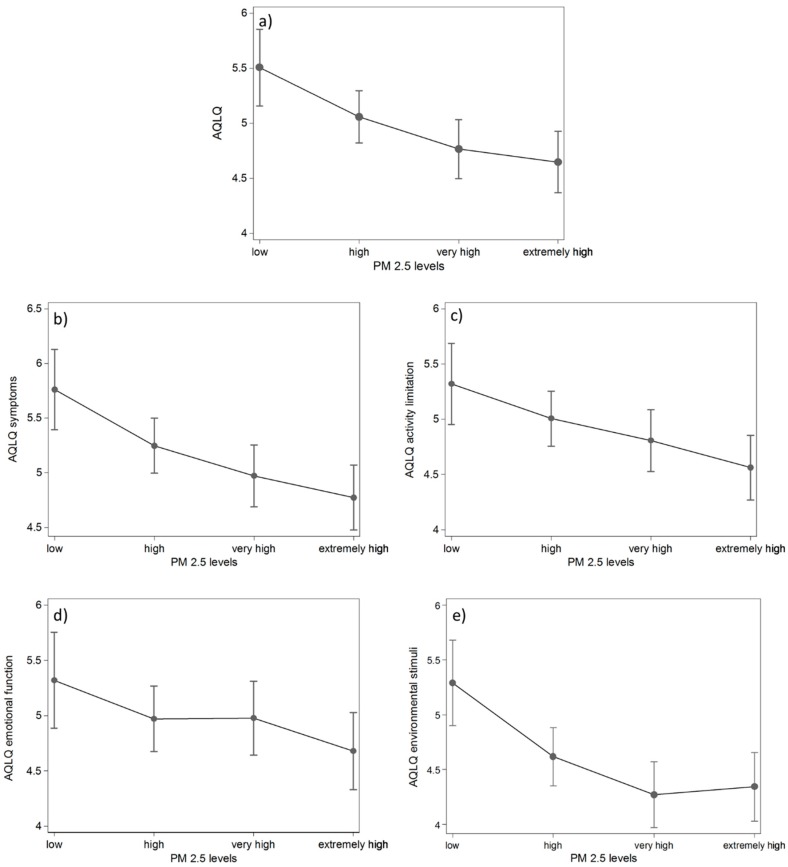
Margins with 95% confidence intervals of (**a**) the Asthma Quality of Life Questionnaire (AQLQ) total, (**b**) AQLQ symptoms, (**c**) activity limitations, (**d**) emotional functioning, and (**e**) environmental stimuli and its domains according to PM_2.5_ levels (linear prediction adjusted to age, gender, active smoking, university education, and season of measurement).

Table 2 shows the effect of categorized PM_2.5_ exposure on asthma quality of life, adjusted to potential confounders. First, we compared patients exposed to the PM_2.5_ values above 25 µg/m^3^ with those exposed to lower pollution levels (≤25 µg/m^3^). A reduction of 0.6 in the AQLQ total was observed in those exposed to the higher levels compared to those with PM_2.5_ exposure ≤25 µg/m^3^, while the deficit in specific domains was statistically significant in the case of AQLQ symptoms and AQLQ environmental stimuli. When quartiles of PM_2.5_ exposure were compared, after adjustments for the confounders, the effect of exposure became significant in all domains (Table 2). The observed effect of the increased level of exposure was associated with reduced values in the AQLQ total in all higher-level quartiles of exposure, in comparison to low-level quartiles. A similar decrease was observed in AQLQ symptoms and AQLQ environmental stimuli domains. In AQLQ activity limitations and emotional functioning, quality of life differed significantly only between the extremely high-level quartile of exposure and low-level quartile. Predicted margins of AQLQ and its domains according to PM_2.5_ levels, categorized by quartile distribution, and adjusted to age, gender, active smoking, university education, and season of measurement, are presented in Figure 1.

To assess the effects of PM_2.5_ exposure on the risk of poorer outcomes in asthma quality of life (total and at specific domains) multivariable ordinal logistic regression models were applied. It was observed that high levels of PM_2.5_ exposure were associated with over two-fold higher risk of poorer outcomes in AQLQ total (OR = 2.68, 95% CI = 1.28; 5.60, *p* = 0.009), while the very high and extremely high exposure were associated with over seven-fold higher (OR = 7.34, 95% CI = 3.50; 15.42, *p* < 0.001 and OR = 7.17, 95%CI = 3.39; 15.20, *p* < 0.001, respectively), compared to the low levels of PM_2.5_ exposure. Similar results were seen in particular domains. The risk of poorer quality of life in AQLQ symptoms reached values from 2.2 times higher for high levels of PM_2.5_ to 5.3 for the extremely high levels compared to the low levels. Higher risk of poor outcomes in AQLQ environmental stimuli was 3.2 times higher for high levels of exposure and 6 times higher for very and extremely high exposure compared to the exposure to low levels (Table 2).

**Figure 2:**
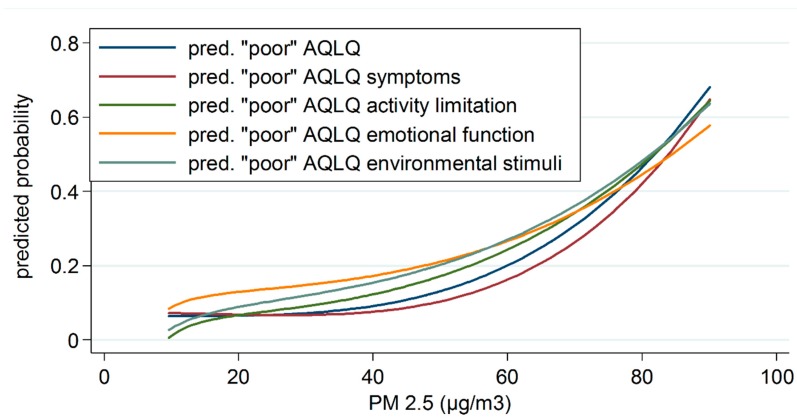
Predicted probabilities of “poor” asthma quality of life (total and for specific domains) according to PM_2.5_ levels.

The effect of a continuously expressed level of PM_2.5_ exposure on asthma quality of life, adjusted to potential confounders, is shown in Table 3. The risk of poorer asthma quality of life increased with higher level of PM_2.5_ exposure. A 10 µg/m^3^ increase in PM_2.5_ was significantly associated with a 46% higher risk of poorer total asthma quality of life (OR = 1.46, 95% CI =1.29; 1.66, *p* < 0.001) after adjustment for age, gender, and university education. Similar results were observed in each domain, where a 10 µg/m^3^ increase in PM_2.5_ increased the risk of poorer outcomes in all AQLQ domains, from 21% in AQLQ emotional functioning to 46% in AQLQ activity limitation (Table 3). Predicted probabilities of “poor” asthma quality of life according to PM_2.5_ exposure in all AQLQ domains are presented in Figure 2.

## 4. Discussion

In our study, we compared the quality of life of patients exposed to various concentrations of environmental PM_2.5_ and we observed that increased levels of PM_2.5_ were related to lower asthma quality of life. Total quality of life and all of its four domains (symptoms, activity limitations, emotional functioning, and environmental stimuli) assessed in the study were significantly reduced as a result of air pollution.

It seems that in evaluating the quality of life it is not just the fact of exceeding the WHO guideline value of PM_2.5_ exposure that is significant, but also the degree of exceeding the WHO guideline value as well—in situations where the WHO guideline value was exceeded multiple times all domains of quality of life show lower scores. The most easily influenced factors of quality of life were symptoms of asthma and environmental stimuli, which decrease with every case of exceeding the WHO guideline value.

The influence of particulate pollutants, including the PM_2.5_ fraction, on different aspects of health has been addressed by many studies. The negative impact of PM_2.5_ on the respiratory system is very well documented by studies, showing connections between concentrations of PM_2.5_ and longevity [19,20], frequency of lung carcinoma [21,22], the incidence of respiratory diseases, and exacerbations of such diseases and cardiovascular diseases [23,24,25].

Mathematical models estimated that exposure to PM_2.5_ has decreased average longevity by 8.6 months in Estonia [19], and other studies documented an increase of longevity by as much as 0.35 years for every 10 µg/m^3^ of reduction in the concentration of this fraction of particles [20]. Long-term exposure to particulate matter PM_2.5_ leads to increased mortality due to lung cancer [26,27] and an increased incidence of circulatory diseases [28]. In the United States of America, overall mortality and morbidity for cardiovascular diseases and pulmonary diseases (including lung cancer) increased by 4%, 6%, and 8% respectively for every additional 10 µg/m^3^ of exposure to PM_2.5_ after excluding other risk factors [29]. It has also been documented that the 15–27% increase in mortality due to lung cancer was connected with the increase in the concentration of PM_2.5_ by 10 µg/m^3^, and the risk was even higher for patients with chronic lung diseases [30]. The results of 11 cohort studies in Europe have shown that the hazard ratio for lung adenocarcinoma was 1.55 for every 5 µg/m^3^ increase in PM_2.5_ [21], and in six regions of Japan, studies have shown a relationship between exposure to increased concentrations of PM_2.5_ and the incidence of lung cancer [22].

The negative impact of PM_2.5_ on the respiratory system is confirmed by a systematic review of epidemiological studies [31]. Other analyses have shown that the increase of PM_2.5_ by 10 µg/m^3^ caused a 2.07% increase in the incidence of respiratory diseases and an 8% increase in the hospitalization rate [32,33]. Among the residents of the Chinese canton Guangzhou, a 12.07% increase in the incidence of respiratory diseases for every 100 µg/m^3^ of increase in PM_2.5_ was described [34].

Air pollution causes oxidative stress, leading to inflammatory responses of respiratory tracts and bronchial hypersensitivity—typical features of asthma [35]. Moreover, it has been proven that long-term exposure to pollutants from motor vehicle exhaust is related to the development of asthma [10,36,37]. Sudden increases in air pollution cause the aggravation of symptoms of asthma and an increase in the number of hospital admissions in both children and adults [10,12,13]. Studies on the influence of PM_2.5_ have demonstrated a relationship between the increased concentration of this fraction of particulate matter and the incidence of new cases of bronchial asthma [38], as well as the deterioration of control, with necessary medical intervention in hospital emergency wards [23,24,39,40].

However, previous studies did not investigate the influences of concentrations of PM_2.5_ on the quality of life of asthma patients. On the one hand, it may seem that widely available diagnostic methods are sufficient to evaluate the control of bronchial asthma, even though they do not reflect a complete evaluation of the patient’s health, which is influenced not only by physical problems but also by psychological and social issues. That is why, on the other hand, the evaluation of the quality of life, especially in chronic diseases, seems to be important, though not always appreciated. It should be emphasized that quality of life is a crucial element of the contemporary understanding of health [41]. Thus, it is important to evaluate various factors that may impact the quality of life. Among such factors are symptoms of illnesses, limitations of activity (physical, social, professional, emotional), and sleep disorders, but also environmental hazards, including particulate pollutants.

Anecdotal evidence suggests that medical doctors specializing in asthma and allergy often register a correlation between a patient’s worse state of being and periods of increased air pollution, but it is difficult to determine whether this is a result of publicity by a media awareness of air pollution, or if the patients’ quality of life is actually decreased at that time. In our study, we recruited patients that were under the regular care of an allergy clinic. It was a fairly homogenous group, that is, patients with partially controlled asthma, well educated about asthma (e.g., recognizing and controlling symptoms of the disease, with knowledge about a type and use of medication) and systematically taking their medication.

Our study had some limitations. We assessed individual exposure based on PM_2.5_ recorded for the city, while more precise methods of evaluating PM_2.5_ exposure are potentially available. For example, patients could carry mobile dust monitors. However, technical and financial limitations related to the equipment and patients’ compliance would make such a study expensive and difficult to conduct, especially on a large group of participants. More sophisticated models could be used to assess personal exposure [42,43,44]. Air quality is changing over space and time and people travel and spend time at various locations. Therefore, in future studies more detailed data should be used, for example, the duration of time spent outside in each location and mode and routes of travel would make it easier to capture the full range of personal exposure.

Moreover, in our models, we used two-week mean values of PM_2.5_ concentration. The aggravation of symptoms of asthma is often caused by sudden increases in air pollution. Therefore, the peak measurements seem more relevant for exacerbations of asthma than the 14 days average. However, it is worth emphasizing that the quality of life of patients with bronchial asthma does not solely depend on acute asthma attacks. Since AQLQ contains questions concerning the last 14 days, we used two-week mean values of PM_2.5_ concentration as an exposure indicator metric. It is also worth noting that since there is no guideline value for a two-week average PM_2.5_ concentration, we used the WHO guideline value, which is a 24 h average value as a limit value. Future studies should also consider other pollutants, since there is growing evidence suggesting the synergistic effect of multi-pollutant exposures [45,46]. Finally, people spend most of their time indoors. For example, our participants spent only 2–3 h a day outdoors, on average. For these reasons, indoor sources of air pollution should be taken into account. However, we have recently shown that outdoor PM_2.5_ concentration strongly predicts indoor concentration [47].

Despite all these limitations, we have been able to show that even relatively simple information about PM_2.5_ concentration, which is easily accessible for citizens, from air quality monitoring stations may predict the quality of life of asthma patients. Being aware that there is no minimum level of safe exposure to PM_2.5_, physicians taking care of asthma patients should pay special attention to the course of illness and control of patients with bronchial asthma to air pollution, especially in places where high concentrations of PM_2.5_ occur during many days over the year.

## 5. Conclusions

Exposure to increased values of PM_2.5_ impacts the quality of life among people with exceptional predispositions, such as bronchial asthma patients, which serves as an important argument in favor of educational activities, especially concerning public health, and raising awareness of the importance of the issue of air pollution.

The deterioration in the quality of life of patients with asthma control and medication suggests the need for special control throughout the illness during periods of exposure to increased concentrations of PM_2.5_, and it is a fact that should be remembered both by doctors and patients themselves.

Besides taking measures for the improvement in the quality of air, it seems equally important to raise awareness about sources of air pollution, factors influencing concentration values, and possible methods of reducing ambient and personal exposure, especially among patients with bronchial hyper-reactivity.

The substantial reduction of air pollution should be the first and immediate request. Appropriate actions should be taken by governmental organizations, local authorities, and inhabitants. Education and raising awareness about the health risk of high-level PM_2.5_ should be a priority for public health experts.

## Figures and Tables

**Table 1:** Characteristics of the study group: all participants and participants divided in four groups based on quartiles of exposure to PM_2.5_ levels.

Characteristics of the Study Group	Total *n* = 300	Low Exposure (≤25 µg/m^3^) *n* = 75	High Exposure (25.1–46 µg/m^3^) *n* = 75	Very High Exposure (46.1–58 µg/m^3^) *n* = 75	Extremely High Exposure (>58 µg/m^3^) *n* = 75	*p*
women	145 (48.3%)	36 (48.0%)	30 (40.0%)	40 (53.3%)	39 (52.0%)	0.356
men	155 (51.7%)	39 (52.0%)	45 (60.0%)	35 (46.7%)	36 (48.0%)	
age (years)	53 ± 15.3	49.8 ± 15.7	51.3 ± 15.5	52.1 ± 13.3	59.0 ± 15.3	0.001
university education	127 (42.3%)	39 (52%)	35 (46.7%)	29 (38.7%)	24 (32%)	0.068
active smoker	31 (10.3%)	1 (1.3%)	11 (14.7%)	16 (21.3%)	3 (4.0%)	<0.001
current employment						
student	11 (3.7%)	3 (4.0%)	4 (5.3%)	3 (4.1%)	1 (1.3%)	0.124
employed	172 (57.5%)	47 (62.7%)	48 (64.0%)	43 (58.1%)	34 (45.3%)	
unemployed	27 (9.0%)	7 (9.3%)	5 (6.7%)	9 (12.2%)	6 (8.0%)	
retired	89 (29.8%)	18 (24.0%)	18 (24.0%)	19 (25.7%)	34 (45.3%)	
missing	1					
keeping pets at home	133 (43.3%)	34 (45.3%)	33 (75%)	28 (37.3%)	38 (50.7%)	0.433
quality of life in asthma						
AQLQ * total	5.0 ± 1.29	5.6 ± 1.11	5.1 ± 1.14	4.8 ± 1.15	4.4 ± 1.43	<0.001
AQLQ symptoms	5.2 ± 1.34	5.7 ± 1.23	5.3 ± 1.13	5.1 ± 1.28	4.6 ± 1.48	<0.001
AQLQ activity limitation	4.9 ± 1.37	5.7 ± 1.14	5.0 ± 1.25	4.7 ± 1.18	4.3 ± 1.51	<0.001
AQLQ emotional function	5.0 ± 1.53	5.6 ± 1.21	5.1 ± 1.36	4.9 ± 1.51	4.4 ± 1.78	<0.001
AQLQ environmental stimuli	4.6 ± 1.33	5.4 ± 1.35	4.6 ± 1.22	4.3 ± 1.22	4.2 ± 1.2	<0.001

* Asthma Quality of Life Questionnaire score.

**Table 2:** Impact of categorized concentration of PM_2.5_ on asthma quality of life (AQLQ) and the risk of poor asthma quality of life, adjusted to potential confounders.

Concentration of PM_2.5_ Categorized According to 24 h Threshold (>25 µg/m^3^, *n* = 225 versus ≤ 25 µg/m^3^, *n* = 75) and According to the Quartiles	Asthma Quality of Life on Original Scale			Categorized Asthma Quality of Life (Ordered from Good as “1” to Poor as “3”)		
	B *	95% CI for B	*p* ^a^	OR	95% CI ** for OR	*p* ^b^
			AQLQ total			
>25 µg/m^3^ versus ≤ 25 µg/m^3^	−0.56	−0.98; −0.13	0.011	5.12	2.72; 9.63	<0.001
high versus low	−0.45	−0.88; −0.01	0.044	2.68	1.28; 5.60	0.009
very high versus low	−0.74	−1.24; −0.24	0.004	7.34	3.50; 15.42	<0.001
extremely high versus low	−0.86	−1.37; −0.34	<0.001	7.17	3.39; 15.20	<0.001
		AQLQ symptoms				
>25 µg/m^3^ versus ≤ 25 µg/m^3^	−0.63	−1.08; −0.17	0.007	3.51	1.82; 6.77	0.001
high versus low	−0.50	−0.97; −0.05	0.029	2.24	1.04; 4.84	0.039
very high versus low	−0.79	−1.32; −0.26	0.003	3.59	1.67; 7.72	0.001
extremely high versus low	−0.99	−1.53; −0.44	<0.001	5.30	2.48; 11.31	<0.001
		AQLQ activity limitation				
>25 µg/m^3^ versus ≤ 25 µg/m^3^	−0.41	−0.86; 0.04	0.074	5.74	3.07; 10.73	<0.001
high versus low	−0.31	−0.77; 0.14	0.177	3.10	1.49; 6.43	0.002
very high versus low	−0.51	−1.03; 0.01	0.055	8.05	3.84; 16.90	<0.001
extremely high versus low	−0.76	−1.30; −0.22	0.006	8.01	3.81; 16.85	<0.001
		AQLQ emotional function				
>25 µg/m^3^ versus ≤ 25 µg/m^3^	−0.38	−0.91; 0.14	0.155	2.30	1.30; 4.07	0.004
high versus low	−0.35	−0.89; 0.19	0.206	1.94	0.98; 3.84	0.055
very high versus low	−0.34	−0.96; 0.28	0.279	2.72	1.36; 5.43	0.004
extremely high versus low	−0.64	−1.28; 0.001	0.050	2.33	1.17; 4.62	0.016
		AQLQ environmental stimuli				
>25 µg/m^3^ versus ≤ 25 µg/m^3^	−0.78	−1.25; −0.30	<0.001	4.78	2.72; 8.38	<0.001
high versus low	−0.67	−1.16; −0.19	0.007	3.24	1.67; 6.26	<0.001
very high versus low	−1.02	−1.58; −0.46	<0.001	6.06	3.04; 12.09	<0.001
extremely high versus low	−0.95	−1.53; −0.37	0.001	6.08	3.02; 12.24	<0.001

* B—standardized coefficient (beta); ** CI— confidence intervals (a) adjusted to age, gender, active smoking, university education and season of measurement; (b) adjusted to age, age^2^, gender, and university education.

**Table 3:** Impact of mean 14-day concentration of PM_2.5_ (increasing unit: 10 µg/m^3^) on asthma quality of life and the risk of poor asthma quality of life, adjusted to potential confounders.

PM_2.5_ Concentration	Asthma Quality of Life on Original Scale			Categorized Asthma Quality of Life (Ordered from Good as “1” to Poor as ”3”)		
	B *	95% CI for B	*p* ^a^	OR	95% CI for OR	*p* ^b^
AQLQ total						
14-day concentrations of PM_2.5_	−0.16	−0.24; −0.07	<0.001	1.46	1.29; 1.66	<0.001
AQLQ symptoms						
14-day concentrations of PM_2.5_	−0.18	−0.27; −0.09	<0.001	1.37	1.20; 1.55	<0.001
AQLQ activity limitation						
14-day concentrations of PM_2.5_	−0.14	−0.23; −0.06	<0.001	1.46	1.29; 1.66	<0.001
AQLQ emotional function						
14-day concentrations of PM_2.5_	−0.15	−0.26; −0.05	0.004	1.21	0.91; 1.20	0.002
AQLQ environmental stimuli						
14-day concentrations of PM_2.5_	−0.14	−0.24; −0.05	0.003	1.40	1.24; 1.58	<0.001

* B—standardized coefficient (beta); (a) adjusted to age, gender, active smoking, university education, and season of measurement; (b) adjusted to age, age^2^, gender, and university education.
